# Does effective therapy of psoriasis prevent the development of psoriatic arthritis?

**DOI:** 10.1016/j.ero.2026.01.005

**Published:** 2026-03-20

**Authors:** Kerem Abacar, Alen Zabotti, Abdulla Watad, Dennis McGonagle

**Affiliations:** 1 Leeds Institute of Rheumatic and Musculoskeletal Medicine, University of Leeds, Chapel Allerton Hospital, Leeds, UK; 2 Rheumatology Institute, Azienda Sanitaria Universitaria del Friuli Centrale, Udine, Italy; 3 The Sackler’s Faculty of Medicine, Tel-Aviv University, Tel-Aviv, Israel; 4 Rheumatology Unit, Tel-Hashomer Medical Centre, Tel-Aviv, Israel; 5 NIHR Leeds Biomedical Research Centre, Leeds Teaching Hospitals NHS Trust, Leeds, UK

## Abstract

Psoriasis frequently precedes psoriatic arthritis (PsA), delineating a tractable window for disease interception. This narrative review integrates mechanistic, imaging, and clinical evidence, together with expert interpretation, to examine whether effective treatment of psoriasis may modify the risk of PsA development. Converging immunopathogenic insights link skin and joint inflammation through interleukin (IL)-23/IL-17–driven pathways, enthesis-synovium crosstalk, and tissue-resident memory T-cell biology. Clinically, progression from psoriasis with arthralgia to PsA occurs in ∼20% of patients within 3 years, typically manifesting with peripheral, synovitis-predominant presentations. Imaging refines short-term risk stratification, with structural entheseal lesions, particularly erosions and new bone formation, representing the most consistent markers of transition. Observational data suggest that biologic therapies used for psoriasis may reduce PsA evolution, with the most consistent evidence for IL-23 pathway blockade. Metabolic factors, notably obesity and weight loss, further modify disease risk, underscoring the role of systemic inflammation and metabolic-immune crosstalk. Together, these findings support the biological plausibility of PsA prevention and justify ongoing interception trials in high-risk psoriasis populations defined by arthralgia and subclinical musculoskeletal inflammation. Robust longitudinal studies integrating biological, imaging, and pragmatic outcomes will be essential to establish causality and define scalable strategies to alter the natural history of psoriatic disease.

## INTRODUCTION AND RATIONALE

There is growing interest in the prevention of autoimmune diseases, including inflammatory arthritis. As psoriasis typically precedes psoriatic arthritis (PsA), intercepting PsA development is an appealing objective [1]. If effective dermatologic therapy for psoriasis also reduces PsA risk, then prevention could be achieved without additional cost or toxicity beyond standard psoriasis care [1]. This places PsA in a unique position compared with other rheumatic disorders. Accordingly, this article is intended as a narrative review that synthesises available evidence with pathophysiological concepts and clinical perspectives.

In rheumatoid arthritis (RA), prevention efforts emphasise identification of synovitis and/or tenosynovitis as the key pathological lesion [2]. Indeed, the volume of tenosynovitis in anti–citrullinated protein antibodies-positive RA is the strongest predictor of RA development [3]. Although tenosynovitis on imaging is common in PsA-related arthralgia [4], clinical and imaging studies suggest that subclinical entheseal abnormalities are associated with an increased risk of subsequent development of clinically overt PsA [4,5]. Nail disease (more closely associated with PsA than with cutaneous psoriasis) constitutes a salient clinical harbinger; in early Leeds observations, ∼90% of PsA cases exhibited nail involvement [6]. Microanatomical studies demonstrate that extensor tendon fibres at the distal interphalangeal joint are intimately anchored to the nail matrix, providing a direct biomechanical conduit from nail to enthesis [7]. Mechanical stress and microtrauma at this interface likely amplify local inflammation. High-resolution computed tomography in humans shows that subclinical enthesopathy is common in psoriasis and predicts subsequent PsA development [8,9].

A recurring clinical question is why synovitis and joint swelling often constitute the initial manifestations of PsA if the disease frequently initiates at entheses. The synovio-entheseal complex (SEC) concept reconciles this apparent paradox: inflammatory signals triggered at relatively avascular entheses can propagate into adjacent, vascular synovial tissues, culminating in soft-tissue swelling and synovitis, which suggests a greater need to properly identify very early forms of PsA, which may likely present with pain but without swelling [10].

## SKIN-JOINT IMMUNOPATHOLOGY: HOW SIMILAR?

Perceived divergence between skin and joint arises primarily from differences in onset timing, as psoriasis commonly precedes arthritis by several years [11,12]. Comparative genomic profiling of paired PsA skin and synovium supports this distinction, demonstrating a dominant interleukin (IL)-17/IL-23 signature in skin but stronger tumour necrosis factor (TNF)- and IL-6-driven programmes in synovium [13]. Nevertheless, inhibitors targeting the IL-23/IL-17 axis achieve excellent skin clearance and also drive resolution of hallmark musculoskeletal features such as dactylitis (often ∼60% at 6 months and ∼80% at 1 year), supporting closer skin-joint immunologic links than previously appreciated [14,15]. Genetic studies reinforce this view: genome-wide association studies consistently highlight IL-23/IL-17 pathway signals among top loci in psoriasis/PsA [[16], [17], [18]].

Recent experimental data have provided compelling evidence that inflammatory processes in psoriatic disease may extend from the skin to the joints through direct immune cell trafficking. Using photoconvertible mouse models, Raimondo et al [19] demonstrated that IL-23-driven skin inflammation is followed by the migration of a distinct population of CD2⁺ major histocompatibility complex-II⁺ CCR2⁺ skin-derived myeloid cells into joint tissues. Importantly, however, the presence of these migrated cells alone was not sufficient to induce arthritis, as joint inflammation failed to develop despite comparable levels of skin-to-joint trafficking in certain contexts. Instead, disease emergence was shown to depend on the local joint microenvironment, particularly the loss of stromal regulatory mechanisms mediated by CD200⁺ fibroblasts [19], thereby defining a permissive inflammatory niche. Although these findings may initially appear to challenge enthesis-centred models of PsA initiation, the 2 frameworks need not be viewed as mutually exclusive. Rather, skin-derived immune cell migration may represent a necessary upstream event, whereas progression to clinically overt arthritis requires a locally conditioned microenvironment at the SEC, shaped by mechanical stress, microdamage, and IL-23-dependent immune priming. Within this integrated view, PsA emerges as a multistep, context-dependent process determined by the convergence of immune cell trafficking and tissue-specific stromal susceptibility.

Translating these genetic signals into tissue-level mechanisms, IL-23R⁺ entheseal T cells have been shown to initiate spondyloarthropathy in relevant experimental models [20]. Normal human enthesis harbours resident myeloid cells capable of constitutive IL-23 production even in the absence of overt inflammation [21]. Human imaging and SKG mouse model data indicate that spinal and axial entheseal sites are particularly rich in neutrophils and IL-23-producing cells, implicating bone-marrow and entheseal niches early in disease evolution [[22], [23], [24], [25]].

Unlike RA, where inflammation often initiates in small metacarpophalangeal joints and tendon sheaths in association with autoantibody-driven synovitis, PsA is more heterogeneous in its tissue origins [26]. Nonetheless, synovitis remains the most common early manifestation, whereas peripheral oligoarthritis typically dominates the initial clinical presentations.

In this context, synovitis should not necessarily be interpreted as definitive evidence of the primary site of disease initiation. Rather, it is apparent prominence may reflect patterns of clinical recognition. Within this framework, proposed links between enthesitis and subsequent synovial involvement remain plausible but unconfirmed and should be considered in light of the marked heterogeneity that characterises PsA [27]. Extra-articular features such as uveitis, inflammatory bowel disease, and nail disease may accompany or even predate musculoskeletal onset, conferring an increased risk of PsA [28,29].

## TISSUE-RESIDENT T CELLS AND CONVERGENT IMMUNOLOGY

Tissue-resident memory T cells (TRM) have been implicated in autoimmune diseases involving both nonbarrier tissues (such as the kidneys in lupus nephritis) and barrier tissues, including the skin in psoriasis and the gut in inflammatory bowel disease [[30], [31], [32], [33]]. Epidermal CD8⁺ TRM are central to psoriasis pathophysiology [34]. IL-23 pathway blockade has been associated with durable cutaneous remission, as shown by the VOYAGE-2 trial withdrawal-retreatment analyses with guselkumab, the randomised GUIDE maintenance trial (every 8 weeks vs every 16 weeks noninferiority to week 68), and long-term risankizumab extension cohorts (LIMMitless open-label extension study, up to ∼5–6 years) [[35], [36], [37]]. Evidence for TRM in synovium and enthesis is also emerging. IL-17-producing TRM-like CD8⁺ T cells have been identified in PsA synovial fluid and tissue, and recent transcriptomic studies highlight TRM signatures within human joint biopsies [38]. A humanised PsA mouse model further demonstrates a CD8-dependent mechanism [39]. Normal human enthesis contains both CD4⁺ and CD8⁺ T cells with TRM phenotypes [40], suggesting a shared tissue-resident compartment across barrier and musculoskeletal sites.

Importantly, TRM are increasingly recognised as enhancers rather than direct effector cells, orchestrating inflammation primarily through the recruitment and activation of other immune and stromal populations [41]. Once activated, synovial TRM are thought to initiate synovitis indirectly by amplifying cytokine networks and recruiting monocytes, macrophages, and fibroblast-like synoviocytes (FLSs), which in turn sustain the inflammatory cascade [41]. This mechanism provides a rationale for considering therapies originally developed for cutaneous disease as potential preventive or interceptive strategies in individuals at risk of developing joint inflammation, as these agents may inhibit the activation of TRM and other enhancer-cell populations involved in early immune amplification [42].

Beyond TRM, FLSs, the predominant stromal cell type within the synovium, may further amplify inflammation through the production of proinflammatory cytokines such as IL-6 and granulocyte–macrophage colony-stimulating factor, whereas both tissue-resident and infiltrating macrophages contribute to the maintenance of the inflammatory milieu [43,44]. Understanding how anti-IL-23 and anti-IL-17 agents modulate these cellular networks, particularly in comparison with TNF inhibitors, is of critical importance, especially given the superior efficacy of IL-17/23 inhibition in cutaneous disease [13,45,46].

## FROM RISK TO TRANSITION: PREDICTORS OF PsA

In RA, progression from at-risk states characterised by inflammatory arthralgia and subclinical synovitis can be predicted with moderate accuracy [2,47]. By contrast, prediction of PsA remains more challenging: among patients with psoriasis with arthralgia, ∼20% progress to PsA within 3 years, most commonly presenting with a peripheral arthritis–predominant phenotype [48]. Imaging studies support this clinical observation, demonstrating SEC inflammation as the dominant subclinical correlate. The relatively low event rate and absence of highly specific enrichment markers make prevention trials difficult to design and adequately power in this population.

Long-term PsA risk factors include nail involvement and extensive plaque psoriasis, together with family history, obesity, and higher disease severity (Psoriasis Area and Severity Index) [1,49]. Short-term predictors include both inflammatory and noninflammatory arthralgia, as well as imaging evidence of subclinical musculoskeletal inflammation [48,50].

The 2024 European Alliance of Associations for Rheumatology Task Force on PsA prevention conceptualised disease evolution as a 3-stage continuum: (i) at-risk psoriasis, (ii) subclinical disease, and (iii) clinical PsA [51]. It is also important to recognise that the transition from psoriasis to PsA is not entirely indeterminate. Although the boundaries between cutaneous disease, subclinical musculoskeletal involvement, and overt arthritis remain blurred, converging imaging and mechanistic data suggest that disease evolution occurs near biologically relevant inflection points. Interception strategies, therefore, operate in the vicinity of these subclinical transition zones, even if their precise definition remains imperfect. Within the subclinical stage, patients presenting with inflammatory-type arthralgia tend to progress more rapidly to clinical PsA, whereas those with mechanical or nonspecific pain remain at elevated risk but typically follow a slower trajectory [48].

Obesity is both a comorbidity and a modifiable risk factor within the psoriatic disease spectrum. Epidemiologic data indicate that obesity confers a 1.5- to 2.5-fold higher risk of PsA development among patients with psoriasis [52], and that weight reduction is associated with lower incidence and milder musculoskeletal expression of disease. These observations underscore the importance of metabolic balance in psoriatic disease interception. Although evidence is still emerging, improvements in metabolic health—including those achieved through glucagon-like peptide-1 (GLP-1) receptor agonists—may ultimately contribute to risk modification, a hypothesis that warrants prospective evaluation.

Beyond its epidemiologic association, obesity acts as a systemic immunometabolic driver of psoriatic inflammation. Adipose tissue functions as an inflamed endocrine organ, producing IL-6, TNF, IL-1β, and leptin, which promote both systemic low-grade inflammation and tissue-specific immune activation. Experimental models demonstrate that a high-fat diet fosters IL-17A-producing γδ T-cell accumulation in the skin and enthesis, whereas chronic lipid overload triggers macrophage infiltration and epigenetic reprogramming of myeloid precursors towards a proinflammatory phenotype, a phenomenon consistent with trained immunity [45]. Moreover, free fatty acids and oxidised lipid species reinforce the metabolic-immune interface by engaging Toll-like receptor and NOD-like receptor family pyrin domain-containing 3 pathways, thereby linking dyslipidaemia with chronic psoriatic inflammation.

In this context, GLP-1 receptor agonists may represent a novel class of disease-modifying agents. In addition to improving insulin sensitivity and promoting weight loss, GLP-1 agonism exerts direct anti-inflammatory effects such as inhibition of nuclear factor kappa B signalling in macrophages, reduced endothelial adhesion-molecule expression, and attenuation of IL-17A and interferon gamma production by T cells, thereby broadening the disease-modifying antirheumatic drug concept to include metabolic interventions that target systemic inflammatory tone and immune-metabolic crosstalk [53] (Figure).

**Figure:**
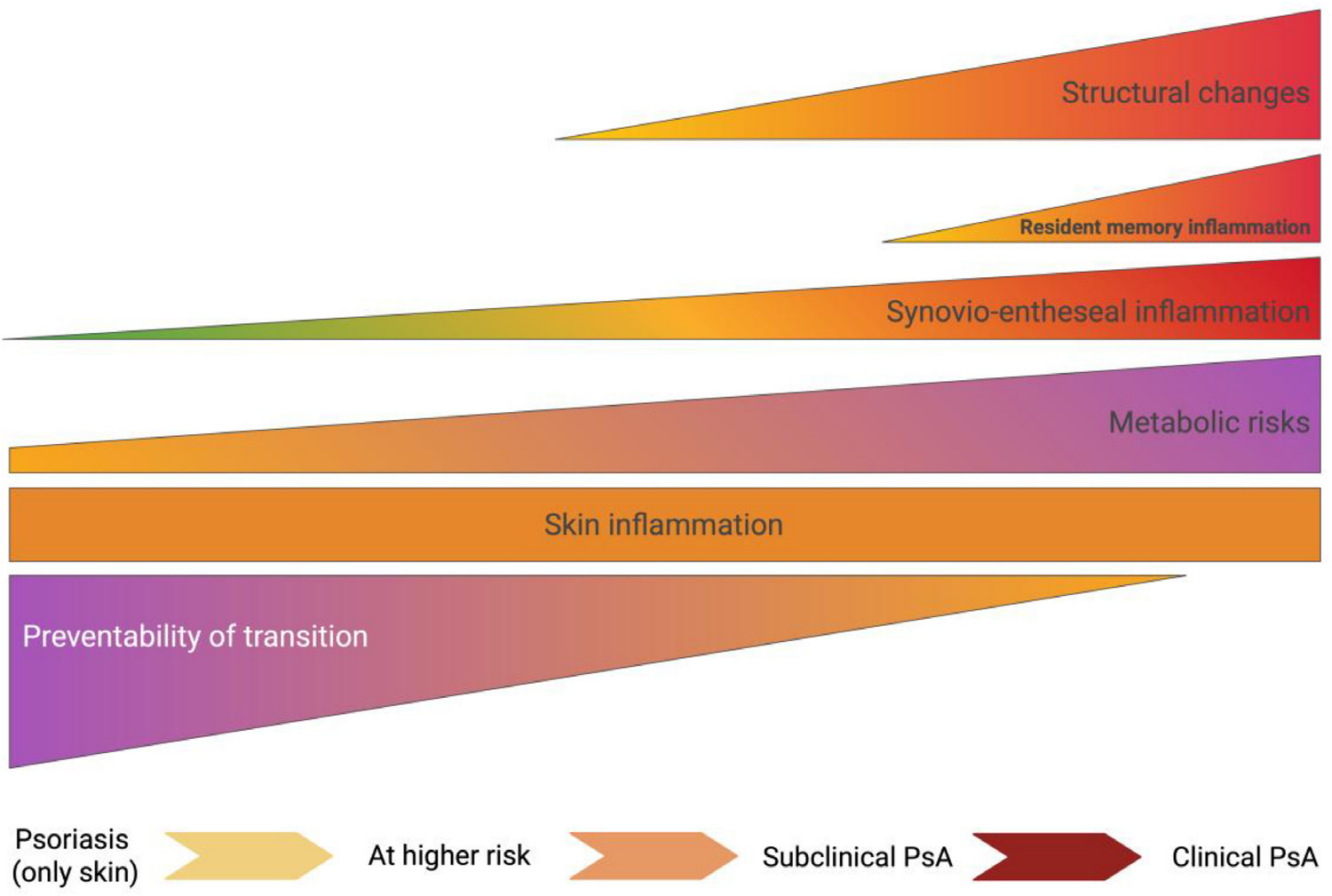
Immunopathologic progression and diminishing preventability across the psoriatic disease spectrum. At higher risk: patients with PsO are at higher risk of PsA (ie, severe skin disease, nail involvement, obesity, familial history). Inflammatory context: systemic IL-23/IL-17 axis activation with predisposing metabolic and genetic factors; no TRM formation yet. Subclinical PsA: patients with PsO with arthralgia and/or imaging evidence of synovial or entheseal inflammation without clinical synovitis. Inflammatory context: early IL-23-driven myeloid activation and emerging TRM establishment at the enthesis; inflammation becomes self-sustaining, evolving towards resident memory inflammation within joint tissues. Clinical PsA: patients with PsO and clinical synovitis. Inflammatory context: stable TRM-stromal-myeloid circuits drive chronic resident memory inflammation and irreversible structural change across the synovio-entheseal complex. IL, interleukin; PsA, psoriatic arthritis; PsO, psoriasis; TRM, tissue-resident memory T cells.

## IMAGING IN THE TRANSITION: STRUCTURAL ENTHESOPATHY AS THE KEY LESION

Imaging is central to understanding the transition from psoriasis to PsA, but interpretation is often complicated by mechanical and metabolic confounders such as age and body mass index (BMI), which themselves influence entheseal morphology. Early imaging studies highlighted the role of the flexor pulley system as an enthesis-related structure frequently thickened in psoriatic subjects [[54], [55], [56]], suggesting that dactylitis and digital pain may arise from pulley enthesitis. Subsequent longitudinal investigations, however, identified structural entheseal lesions, particularly erosions and new bone formation, as the imaging features most consistently associated with PsA development [4,9].

In the multicentre ultrasound study by Zabotti et al [4], baseline entheseal inflammation and erosive changes were independently associated with subsequent PsA onset, supporting the view that the enthesis represents the primary anatomical site of transition. In the longitudinal magnetic resonance imaging (MRI) study by Simon et al [9], structural entheseal lesions, particularly erosions and new bone proliferation, were predictive of future PsA, whereas transient inflammatory changes alone were not. Together, these findings establish structural enthesopathy as the most consistent imaging marker of PsA transition, reflecting cumulative biomechanical stress and chronic inflammation within the SEC. Although interpretation may be confounded by age, BMI, and mechanical load, these convergent studies support a pathogenic model in which PsA arises from localised entheseal injury that propagates into adjacent synovium and bone marrow, marking a distinct evolutionary pathway from RA.

## DOES EFFECTIVE PSORIASIS THERAPY PREVENT PsA?

Ultrasound studies indicate that ustekinumab can regress subclinical entheseal abnormalities in psoriasis without clinical PsA [57]; similar regression has been reported with secukinumab in patients with subclinical musculoskeletal inflammation [58]. MRI-based observational datasets and large electronic medical records cohorts have subsequently expanded this field. Earlier network signals (eg, TriNetX) suggesting an apparent increase in PsA under TNF inhibitors are likely explained by protopathic bias and surveillance differences. More recent longitudinal analyses do not support an increased PsA risk with TNF blockade; if anything, several cohorts suggest a neutral to protective association. Multiple real-world analyses report lower PsA incidence under dermatologic biologics, although causality cannot be inferred from these designs [59].

Across datasets, an emerging pattern is that IL-23 inhibitors may associate with a lower risk of PsA onset relative to other mechanisms, including TNF inhibitors [[60], [61], [62]]. Conversely, prior exposure to multiple distinct biologics in psoriasis cohorts correlates with higher subsequent PsA risk, most plausibly reflecting refractory, systemically active skin disease and channelling/surveillance biases rather than a direct drug effect. These observations are hypothesis generating and require rigorous confounder control (including disease severity, treatment history, and healthcare utilisation) and replication in prospective settings.

## MECHANISTIC SYNTHESIS AND TRIAL DESIGN

Mechanistic convergence, IL-23/IL-17 circuits, enthesis-synovium crosstalk, and TRM biology support the plausibility that effective dermatologic therapy could reduce PsA evolution [20,21,38,40,63]. Many psoriasis biologics have favourable long-term safety and treatment persistence, making them attractive candidates for interception strategies [37,64]. Unlike RA, prevention in PsA can be explored not only in symptomatic arthralgia cohorts but also in asymptomatic psoriasis enriched by risk markers (eg, nail disease, obesity, family history, and imaging-defined entheseal lesions).

Ongoing and proposed studies are testing whether initiating an IL-23 inhibitor for skin disease in high-risk psoriasis (eg, inflammatory-type arthralgia plus subclinical SEC inflammation) lowers progression to clinical PsA compared with optimised dermatologic care [65]. As a case in point, Preventing Arthritis in a Multicentre Psoriasis At-Risk cohort is designed to randomise patients with either severe psoriasis or psoriasis at sensitive sites, who also have peripheral arthralgia and prespecified ultrasound abnormalities, to guselkumab vs optimised dermatologic standard of care. Primary outcomes focus on improvement in musculoskeletal symptoms and prevention of transition to clinical PsA. (Protocol citation pending; Preventing Arthritis in a Multicentre Psoriasis At-Risk cohort framework referenced in [65]).

## HOW TO DEFINE CLINICAL PSA: LIMITATIONS IN CLINICAL PRACTICE AND CLINICAL TRIALS

Defining the onset of clinical PsA remains a major challenge for both clinicians and trialists. The Classification Criteria for Psoriatic Arthritis classification criteria [66] although reliable for established disease, lack sensitivity for very early or enthesitis-predominant presentations. This limitation, compounded by the inherently heterogeneous nature of PsA and its variable modes of onset, contributes to diagnostic delay and under-recognition of early disease. The role of imaging further complicates this landscape: although ultrasound and MRI can reveal subclinical inflammation, it remains uncertain which lesions mark the true transition to definite arthritis, even in axial forms [51]. Collectively, these challenges underscore the need for harmonised definitions that integrate clinical, serological, and imaging domains to more precisely delineate the point of transition from psoriasis to PsA.

## CONCLUSIONS

PsA evolves through an enthesis-centred pathway linking skin and joint inflammation. Imaging studies highlight structural entheseal lesions—erosions and new bone formation—as the most consistent markers of transition, distinguishing PsA from synovitis-driven diseases such as RA. Mechanistic and clinical observations implicate the IL-23/IL-17 axis as a key driver, with IL-23 blockade among the most promising candidates for prevention. Metabolic factors such as obesity further amplify disease risk, suggesting that metabolic modulation and immune interception may be complementary approaches. Collectively, these insights support the biological plausibility of PsA prevention, warranting confirmation in longitudinal and interventional studies integrating immunology, imaging, and clinical outcomes.

## CRediT authorship contribution statement

**Kerem Abacar:** Writing – review & editing, Writing – original draft, Data curation. **Alen Zabotti:** Writing – original draft, Supervision, Methodology, Investigation, Conceptualization. **Abdulla Watad:** Writing – review & editing, Supervision, Conceptualization. **Dennis McGonagle:** Writing – review & editing, Writing – original draft, Visualization, Validation, Supervision, Resources, Methodology, Investigation, Data curation, Conceptualization.
